# A Challenging Case of Adult-Onset Still's Disease Complicated by Macrophage Activation Syndrome With Multiorgan Failure

**DOI:** 10.7759/cureus.43128

**Published:** 2023-08-08

**Authors:** Abdelaziz Mohamed, Ahmed K. A. Yasin, Anas Mohamed, Nusiba Elamin, Salma Mustafa, Abdalla Fadul, Ghulam Syed, Mustafa Al-Tikrity

**Affiliations:** 1 Internal Medicine, Hamad Medical Corporation, Doha, QAT; 2 Molecular Imaging and Nuclear Medicine, Hamad Medical Corporation, Doha, QAT; 3 Pulmonology, Hamad Medical Corporation, Doha, QAT

**Keywords:** adult-onset still's disease, connective tissue diseases, inflammatory cytokines, macrophage activation syndrome, hemophagocytic lymphohistiocytosis

## Abstract

Adult-onset Still's disease (AOSD) is a rare systemic inflammatory disease that involves the excessive production of proinflammatory cytokines. Hemophagocytic lymphohistiocytosis (HLH) is a life-threatening immune-mediated disorder that can be primary or secondary to malignancy, infections, and autoimmune diseases. We present an interesting case of a young female with adult-onset Still's disease that commenced during pregnancy. Whole-body fluorodeoxyglucose (FDG)-positron emission tomography (PET)/computed tomography (CT) scan showed diffuse uptake in the spleen and bone marrow with widespread lymphadenopathy. During the delayed diagnostic process to exclude lymphoproliferative malignancy, she developed severe HLH/macrophage activation syndrome (MAS) with multiorgan failure. In this case report, we described the challenges faced during the diagnosis of AOSD. We also highlighted the importance of using clinical criteria to aid in the early diagnosis and management of AOSD patients and the role of FDG-PET/CT scans in patients with AOSD. Additionally, we discussed the management aspects for patients with macrophage activation syndrome.

## Introduction

Adult-onset Still's disease (AOSD) is a systemic inflammatory disease of unclear etiology. The excessive production of proinflammatory cytokines, including interleukins, interferon-γ (IFN-γ), and tumor necrosis factor-α (TNF-α), plays a significant role in the pathogenesis of AOSD [1]. Pregnancy is a known trigger for the onset of AOSD with around 20 cases reported in the literature [2]. Hemophagocytic lymphohistiocytosis (HLH) is a life-threatening immune-mediated disorder. The uncontrolled proliferation and activation of macrophages and lymphocytes in patients with HLH leads to widespread systemic inflammation. HLH can be primary, mainly seen in infants and children, or secondary to malignancy, autoimmune diseases, or chronic infections [3]. When it is associated with autoimmune diseases, HLH is also called macrophage activation syndrome (MAS) [3]. The most common infectious trigger for HLH is Epstein-Barr virus (EBV), which was observed in one-third of HLH cases [4]. Macrophage activation syndrome (MAS) was reported in 15% of AOSD patients, and it confers a high risk of mortality [5]. In this case report, we describe an interesting case of a young female diagnosed with AOSD during pregnancy. A few months after delivery, she developed severe EBV-associated MAS/HLH with multiorgan failure.

## Case presentation

A 37-year-old female with a medical background of thalassemia trait and iron deficiency anemia came to our emergency department with persistent high-grade fever. Her symptoms started one year before admission with bilateral inguinal lymphadenopathy when she was 26 weeks pregnant. A few weeks later, she developed intermittent high-grade fever, joint pain mainly involving hand joints, and erythematous skin rash involving the abdomen and both thighs. She was assessed initially in another center, and an Inguinal lymph node biopsy at that time revealed hyperplastic lymph nodes with no features of malignancy. She was diagnosed with possible AOSD and started on hydroxychloroquine and oral prednisolone.

**Figure 1:**
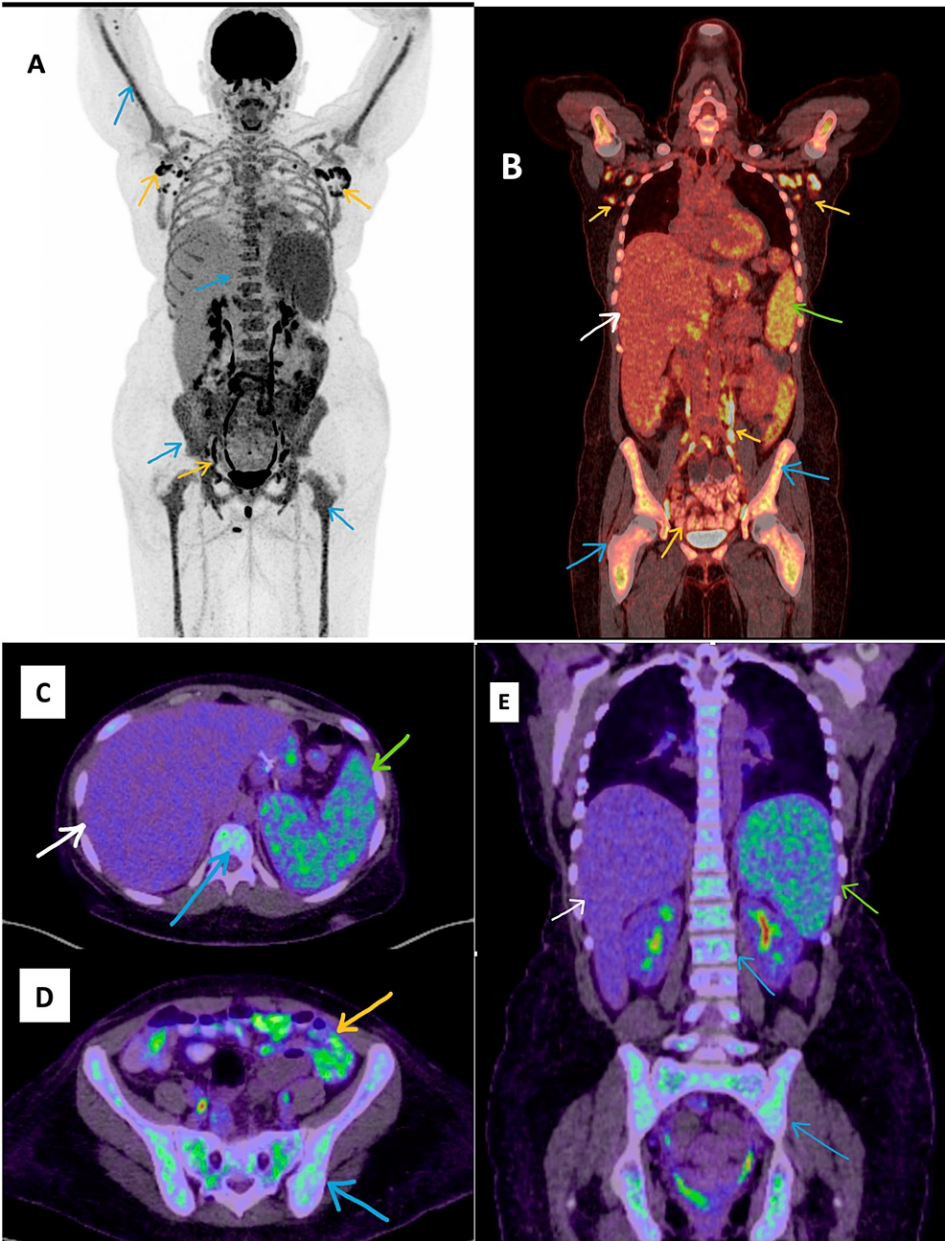
FDG-PET/CT scan with MIP image (A), fused coronal images (B,E), and fused axial images (C,D) Multiple hypermetabolic lymph nodes in the bilateral axillary, inguinal, iliac, para-aortic, aortocaval, and retroperitoneal regions (yellow arrows in A, B, and D). Marked hepatosplenomegaly with intense uptake in the spleen in B, C, and E (spleen shown as green arrows and liver shown as white arrows). Intense FDG uptake in the bone marrow, vertebra, and bilateral femurs and humerus in all images (blue arrows). FDG-PET/CT: fluorodeoxyglucose-positron emission tomography/computed tomography, MIP: maximum intensity projection

Six months later, she visited our hospital clinic complaining of worsening fever, sore throat, and night sweating. She was still taking oral prednisolone, and it has only improved her joint pain, but she continued to have a high-grade fever. Workup for common infectious diseases, including TB, *Brucella*, and HIV was negative. Complete investigations for autoimmune antibodies were also negative, including antinuclear antibodies and rheumatoid factor. Further laboratory investigations showed high ferritin (6,150 ug/L) and high WBC (11.3 × 10^3^/uL) with granulocyte predominance. Basic investigations and investigations for infectious and autoimmune diseases are summarized in Table 1. The abdominal US showed hepatosplenomegaly. The patient was diagnosed with AOSD according to Yamaguchi criteria. She fulfilled all four major criteria and four out of the five minor criteria. A whole-body positron emission tomography (PET)/computed tomography (CT) scan was done to investigate for possible malignancy, which revealed multiple slightly enlarged hypermetabolic generalized lymphadenopathy and marked hepatosplenomegaly with intense uptake in the spleen and bone marrow (Figure 1). Because of the concerning findings in the PET/CT scan, an inguinal lymph node excisional biopsy was done and showed reactive lymphoid follicular hyperplasia and polyclonal plasmacytosis with no features suggestive of lymphoma or malignant process. During this time, her oral prednisolone was tapered off, and she was planned for a bone marrow biopsy to rule out hematologic malignancies.

**Table 1:** Initial laboratory investigations before admission WBC: white blood cell, MCV: mean cell volume, INR: international normalized ratio, APTT: activated partial thromboplastin time, AST: aspartate transaminase, ALT: alanine aminotransferase, ALP: alkaline phosphatase, LDH: lactate dehydrogenase, CRP: C-reactive protein, anti-dsDNA Ab: anti-double-stranded DNA antibodies, anti-CCP Ab: anti-cyclic citrullinated peptide antibodies, ANCA: antineutrophil cytoplasmic antibody, ANA: antinuclear antibody, RNP: ribonucleoprotein antibody, AFB: acid-fast bacilli, TB: tuberculosis, HIV: human immunodeficiency virus, COVID-19: coronavirus disease 2019, PCR: polymerase chain reaction, EBV: Epstein-Barr virus

Variable	Measurement	Reference values
WBC	8.6 × 10^3^/uL	4-10 × 10^3^/uL
Neutrophils	7.1 × 10^3^/uL	2-7 × 10^3^/uL
Lymphocytes	1.4 × 10^3^/uL	1-3 × 10^3^/uL
Hemoglobin	9.1 gm/dL	12-15 gm/dL
MCV	68.7 fL	83-101 fL
Platelets	509 × 10^3^/uL	150-410 × 10^3^/uL
Prothrombin time	14.6 seconds	9.4-12.5 seconds
INR	1.4	-
APTT	31.7 seconds	25.1-36.5 seconds
Urea	2.3 mmol/L	2.5-7.8 mmol/L
Creatinine	56 umol/L	44-80 umol/L
Sodium	136 mmol/L	133-146 mmol/L
Potassium	5.2 mmol/L	3.5-5.5 mmol/L
Bicarbonate	26 mmol/L	22-29 mmol/L
Calcium	2.20 mmol/L	2.20-2.60 mmol/L
Total bilirubin	4 umol/L	0-21 umol/L
Albumin	25 gm/L	35-50 gm/L
ALP	65 U/L	35-104 U/L
ALT	8 U/L	0-33 U/L
AST	39 U/L	0-32 U/L
LDH	568 U/L	135-214 U/L
CRP	143.6 mg/L	0-5 mg/L
Ferritin	6,150 ug/L	12-160 ug/L
Anti-dsDNA Ab	Negative	
Rheumatoid factor	Negative	
Anti CCP Ab	Negative	
ANCA	Negative	
ANA	Negative	
Complements C3, C4	Normal	
RNP	Negative	
RNP 70	Negative	
RO52	Negative	
AFB smear, culture	Negative	
Quantiferon TB	Negative	
*Brucella* antibodies	Negative	
Blood culture	Negative	
Urine culture	Negative	
HIV Ag/Ab	Negative	
COVID-19 PCR	Negative	
Hepatitis B sAg	Negative	
EBV IgG	Positive	
EBV IgM	Negative	

Two weeks after stopping prednisolone therapy, she presented to the emergency department complaining of high-grade fever not responding to antipyretics, significant fatigue, and worsening arthralgia. Vitals signs showed temperature reaching 39.5°C, BP of 87/56 mmHg, and heart rate reaching 137 BPM. On the second day of admission, she was transferred to the medical intensive care unit (ICU) due to worsening hypotension not responding to intravenous fluids. She was started on broad-spectrum antibiotics and required a high dose of noradrenaline to maintain her blood pressure. Upon admission to the intensive care unit, her investigations revealed significantly elevated inflammatory markers with high ferritin reaching 98,422 ug/L. EBV PCR was positive (814 IU/mL). Bone marrow aspiration and biopsy were done. Blood culture, urine culture, and full viral panel, including COVID-19 PCR, were all negative for infectious causes. Detailed blood investigations done on day 2 are summarized in Table 2.

**Table 2:** Laboratory investigations upon admission to the intensive care unit WBC: white blood cell, Hgb: hemoglobin, MCV: mean cell volume, PT: prothrombin time, INR: international normalized ratio, APTT: activated partial thromboplastin time, AST: aspartate transaminase, ALT: alanine aminotransferase, ALP: alkaline phosphatase, LDH: lactate dehydrogenase, CRP: C-reactive protein, EBV: Epstein-Barr virus, PCR: polymerase chain reaction, RSV: respiratory syncytial virus, MERS: Middle East respiratory syndrome

Variable	Measurement	Reference range
WBC	37.8 × 10^3^/uL	4-10 × 10^3^/uL
Neutrophils	34.5 × 10^3^/uL	2-7 × 10^3^/uL
Hgb	6.4 gm/dL	12-15 gm/dL
MCV	66.7 fL	83-101 fL
Platelet	138 × 10^3^/uL	150-410 × 10^3^/uL
INR	1.7	-
D-dimer	21.60 mg/L	0-0.49 mg/L
Fibrinogen	2.39 gm/L	2-4.10 gm/L
Urea	6.7 mmol/L	2.5-7.8 mmol/L
Creatinine	160 umol/L	44-80 umol/L
Sodium	127 mmol/L	133-146 mmol/L
Potassium	3.5 mmol/L	3.5-5.5 mmol/L
Bicarbonate	13 mmol/L	22-29 mmol/L
Phosphorus	1.92 mmol/L	0.80-1.50 mmol/L
Total bilirubin	4 umol/L	0-21 umol/L
ALP	238 U/L	35-104 U/L
ALT	6 U/L	0-33 U/L
AST	76 U/L	0-32 U/L
Albumin level	15 gm/L	35-50 gm/L
Triglyceride	2.1 mmol/L	-
IgG	20.25 gm/L	7-16 gm/L
LDH	1,690 U/L	135-214 U/L
CRP	325.7 mg/L	0-5 mg/L
Ferritin	98,422 ug/L	12-160 ug/L
Procalcitonin	37.20 ng/mL	-
Blood culture	Negative	
Urine culture	Negative	
Fungal culture	Negative	
EBV PCR	814 IU/mL (positive)	
RSV	Negative	
Influenza virus A PCR	Negative	
Influenza PH1N1	Negative	
Influenza A H3 virus	Negative	
Influenza A H1 virus	Negative	
Influenza virus B PCR	Negative	
Coronavirus 229E PCR	Negative	
Coronavirus OC43 PCR	Negative	
Coronavirus HKU PCR	Negative	
Parainfluenza virus PCR	Negative	
Human metapneumovirus PCR	Negative	
Bocavirus PCR	Negative	
*Mycoplasma pneumoniae* PCR	Negative	
Bordetella pertussis	Negative	
Legionella pneumophila	Negative	
MERS coronavirus	Negative	
Human rhinovirus	Negative	
Human enterovirus	Negative	

On day 3 of admission, her overall status was deteriorating. She continued to spike high-grade fever and developed tachypnea and desaturation. Repeated investigations were suggestive of acute kidney injury and hepatotoxicity, indicating multiorgan failure. Because of the background of AOSD and negative cultures, HLH/MAS was suspected. According to Khare et al., the H score performs better than the HLH 2004 criteria for predicting the likelihood of HLH [6]. The calculated H score for our patient was 248 points, indicating >90% likelihood of HLH.

**Figure 2:**
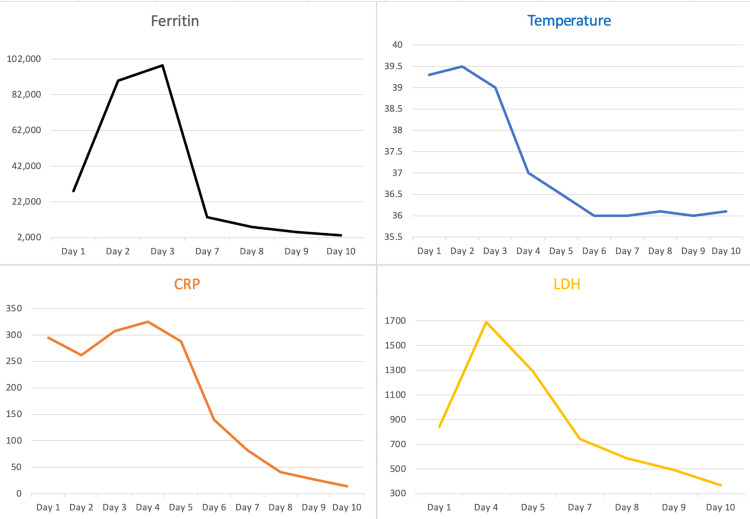
Ferritin, temperature, CRP, and LDH levels before and after IV methylprednisolone given on day 3 Dramatic improvement in all parameters is observed. CRP: C-reactive protein, LDH: lactate dehydrogenase, IV: intravenous

Due to the critical situation of her condition, she was started on IV methylprednisolone 250 mg daily. We verbally contacted the hematopathology laboratory for preliminary results of the bone marrow aspiration and biopsy, and they confirmed the presence of hemophagocytic activity. The patient was started on high-dose dexamethasone as a part of the HLH treatment protocol. Etoposide was offered, but the patient preferred not to take chemotherapeutic agents. Over the next two days, we noticed a dramatic improvement in her condition. Her fever subsided completely, her BP improved, and we were able to wean her off inotropes. Inflammatory markers, including ferritin, have also improved significantly, as shown in Figure 2. After about 10 days of the procedure, the final report of the bone marrow aspiration and biopsy revealed cellular bone marrow with granulocytic hyperplasia, increased plasma cells, and hemophagocytosis with no evidence of blast cells. She was observed on the medical floor for another two weeks, after which she was discharged with no complications. The patient is currently maintained on tocilizumab and oral steroids for her AOSD.

## Discussion

We report a case of AOSD that started during pregnancy and later developed severe HLH/MAS with multiorgan failure. The diagnosis of AOSD can be challenging because of the similar clinical presentation to other common illnesses, e.g., infectious diseases, malignancy, and other autoimmune diseases. Since AOSD is a diagnosis of exclusion, definitive diagnosis and hence proper treatment can be delayed, which may lead to life-threatening complications, including HLH/MAS, diffuse alveolar hemorrhage, acute respiratory distress syndrome, disseminated intravascular coagulopathy, and thrombotic thrombocytopenic purpura [7,8]. Thus, early diagnosis and treatment of AOSD may help prevent such complications. Our patient developed the first manifestations of AOSD more than one year before the diagnosis was confirmed.

FDG-PET/CT scan has an important role in patients with AOSD as it helps measure the level of systemic involvement and identify the suitable site for lymph node or bone marrow biopsy [9]. A total metabolic lesion volume (MLV) of lymph nodes of more than 62.2 is considered to be a strong predictor for the development of MAS in patients with AOSD [10]. A study in 2017 reviewed the FDG-PET/CT scan for 32 patients with confirmed AOSD [9]. Although most patients demonstrated significant uptake in the spleen along with diffuse bone marrow and lymph node involvement, those features are considered nonspecific. Hence, the diagnosis of AOSD should be based on clinical criteria whenever AOSD is suspected. The Yamaguchi criteria were introduced in 1992 for the diagnosis of AOSD [11]; the major criteria include fever, arthralgia, typical rash, and leukocytosis. The minor criteria include sore throat, lymphadenopathy, and/or splenomegaly, abnormal liver function, and negative rheumatoid factor and antinuclear antibody. The finding of five or more criteria, including two or more major criteria, signifies 92.1% specificity and 96.2% sensitivity for the diagnosis of AOSD.

The incidence of AOSD during pregnancy is rare; most reported cases occurred during the first and second trimesters [2]. A review of 19 reported cases revealed that AOSD could result in prematurity and intrauterine growth restriction, which might require intravenous corticosteroids or intravenous immunoglobulin in case of life-threatening AOSD [2]. Our patient developed AOSD manifestations during the third trimester of pregnancy. She had a normal vaginal delivery with no fetal complications.

According to Efthimiou et al. (2014), macrophage activation syndrome occurs in 12%-14% of AOSD cases with a mortality rate of 10%-20%. In addition, the triggering factors for MAS in AOSD patients include disease flare, infections, and sometimes medications. Reactivation of latent viruses such as Epstein-Barr virus or cytomegalovirus might occur because of the low immunity state induced by AOSD treatment, which could also trigger MAS in AOSD patients [12]. Our patient had documented an EBV infection during her presentation, which could trigger MAS. Another possible trigger is a flare of AOSD, as she presented with worsening symptoms two weeks after oral steroids were tapered off and then developed MAS. This might suggest that keeping AOSD patients on steroids while confirming the diagnosis and excluding other diseases might help prevent disease flare and reduce the risk of developing life-threatening complications. The diagnosis of HLH can be challenging because it can present similar to septic shock. HLH and septic shock can overlap, and early diagnosis and management are crucial as both conditions can lead to multiorgan failure and death [13]. Although our patient was initially managed as a case of sepsis and septic shock, the negative blood and urine cultures and the underlying diagnosis of AOSD have raised the possibility of HLH.

Khare et. al (2021) described the performance of the H score to predict the likelihood of HLH. The authors mentioned different clinical and laboratory criteria that are included in the H score. The clinical parameters are the temperature, the presence or absence of immunosuppression, and/or organomegaly. The laboratory parameters include the presence of hemophagocytosis features on bone marrow aspirate, the levels of ferritin, triglycerides, fibrinogen, and serum glutamic oxaloacetic transaminase, and the number of cell lineages with cytopenia (anemia, leukopenia, and/or thrombocytopenia) [6]. Using the H score in our patient has allowed the early diagnosis of HLH without waiting for the results of the bone marrow biopsy. The early initiation of intravenous corticosteroids was a crucial step in managing our patient, which led to a favorable outcome.

## Conclusions

In conclusion, we presented a young female with AOSD that manifested during pregnancy. The delayed diagnosis of her condition has contributed to the development of severe MAS/HLH with multiorgan failure. Although it is important to exclude other differential diagnoses, e.g., lymphoproliferative malignancy, the delayed diagnosis and treatment of AOSD might lead to disease progression and the development of life-threatening complications, e.g., macrophage activation syndrome. Diagnostic clinical criteria, e.g., Yamaguchi criteria, can be used to diagnose AOSD cases while excluding other differential diagnoses. PET/CT scan has nonspecific findings and does not aid in diagnosing AOSD, but it helps determine the extent of the disease and predict the development of MAS. The early introduction of corticosteroids in patients with MAS can lead to rapid recovery and reduce mortality risk.

## References

[1] Maranini B, Ciancio G, Govoni M (2021). Adult-onset Still’s disease: novel biomarkers of specific subsets, disease activity, and relapsing forms. Int J Mol Sci.

[2] Plaçais L, Mekinian A, Bornes M, Poujol-Robert A, Bigé N, Maury E, Fain O (2018). Adult onset Still's disease occurring during pregnancy: case-report and literature review. Semin Arthritis Rheum.

[3] Panagopoulos P, Katsifis G (2018). Secondary hemophagocytic lymphohistiocytosis in a patient with rheumatoid arthritis and vasculitis: a case report and review of the literature. Mediterr J Rheumatol.

[4] Marsh RA (2017). Epstein-Barr virus and hemophagocytic lymphohistiocytosis. Front Immunol.

[5] Giacomelli R, Ruscitti P, Shoenfeld Y (2018). A comprehensive review on adult onset Still's disease. J Autoimmun.

[6] Khare N, Jinkala SR, Kanungo S (2021). Performance of HScore in reactive hemophagocytic lymphohistiocytosis. Indian J Hematol Blood Transfus.

[7] Efthimiou P, Yadlapati S (2018). Adult-onset Still’s disease. Auto-Inflammatory Syndromes.

[8] Kadavath S, Efthimiou P (2015). Adult-onset Still's disease-pathogenesis, clinical manifestations, and new treatment options. Ann Med.

[9] Jiang L, Xiu Y, Gu T, Dong C, Wu B, Shi H (2017). Imaging characteristics of adult onset Still's disease demonstrated with 18F-FDG PET/CT. Mol Med Rep.

[10] Wan L, Gao Y, Gu J (2021). Total metabolic lesion volume of lymph nodes measured by (18)F-FDG PET/CT: a new predictor of macrophage activation syndrome in adult-onset Still's disease. Arthritis Res Ther.

[11] Yamaguchi M, Ohta A, Tsunematsu T (1992). Preliminary criteria for classification of adult Still's disease. J Rheumatol.

[12] Efthimiou P, Kadavath S, Mehta B (2014). Life-threatening complications of adult-onset Still's disease. Clin Rheumatol.

[13] Hindi Z, Khaled AA, Abushahin A (2017). Hemophagocytic syndrome masquerading as septic shock: an approach to such dilemma. SAGE Open Med Case Rep.

